# Exploration of novel harm reduction approaches to increase client engagement (ENHANCE): protocol for a prospective cohort study

**DOI:** 10.1186/s12954-025-01212-0

**Published:** 2025-05-19

**Authors:** Rachel E. Gicquelais, Caitlin J. Conway, Mikaela Becker, Erika J. Bailey, Cullen Bosworth, Rebecca Miller, Katy Mijal, Emmie Curran, Bianca Barredo, Sydney Taylor, Elizabeth Salisbury-Afshar, David W. Seal, Marguerite Burns, Ryan P. Westergaard

**Affiliations:** 1 Department of Population Health Sciences, University of Wisconsin-Madison School of Medicine and Public Health, 610 Walnut Street, Madison, WI 53726 USA; 2 Department of Medicine, Division of Infectious Disease, University of Wisconsin-Madison School of Medicine and Public Health, 610 Walnut St, Madison, WI 53726 USA; 3 University of Wisconsin-Madison School of Nursing, 701 Highland Ave, Madison, WI 53705 USA; 4 Vivent Health, 1311 N 6 Th St, Milwaukee, WI 53212 USA; 5 Department of Family Medicine and Community Health, University of Wisconsin-Madison School of Medicine and Public Health, 610 N Whitney Way, Madison, WI 53705 USA; 6 Tulane University School of Public Health and Tropical Medicine, 1440 Canal Street, New Orleans, LA 70112 USA

**Keywords:** Overdose, Substance use, Study protocol, Prospective cohort study, Community-engaged research

## Abstract

**Background:**

Syringe services programs (SSPs) reduce the risk of overdose by distributing supplies like naloxone. SSPs also support clients in meeting their basic needs via referrals to organizations providing food, housing, and healthcare. This paper describes the Exploration of Novel Harm Reduction Approaches to Increase Client Engagement (ENHANCE) Project, a prospective cohort study developed in partnership with people who use drugs that aims to characterize the influence of longitudinal engagement in SSPs on overdose risk behaviors.

**Methods:**

The ENHANCE Project protocol was developed with a community leadership team of 16 people with living experience of drug use who were clients from four SSPs in Wisconsin that serve as study recruitment sites. The community leadership team met five times to conceptualize the study priorities, protocol, recruitment strategies, and measures. ENHANCE will enroll 400 people who use opioids and/or stimulants via recruitment of clients from study sites and peer referrals. Clients will report on primary outcomes (overdose experiences and risk behaviors) and other covariates (substance use history and behaviors, mental and physical health, satisfaction of basic needs, stigma, and others) over a 2-year follow-up period. At enrollment, clients select a self-generated identification code that is documented in all subsequent harm reduction services received from ten SSPs in Wisconsin. These data will be linked to study survey data using probabilistic methods and used for the primary exposure variable, frequency of using SSP services. ENHANCE will test the primary hypothesis that more frequently using SSP services is associated with reduced overdose risk behavior frequency.

**Preliminary results:**

Among the first 125 clients enrolled, 22.4% (*N* = 28) reported personally experiencing an opioid overdose in the 6 months before enrollment and 38.4% (*N* = 48) experienced adverse effects after using stimulants. The most common overdose risk behaviors reported in the past 30 days were using opioids while alone (mean: 9.9 days [standard deviation: 10.7]) and injecting heroin (mean: 9.3 days [standard deviation: 12.8]).

**Conclusions:**

This study will identify aspects of harm reduction services that are most effective in reducing overdose risk to inform future service provision, funding, and policy efforts.

**Supplementary Information:**

The online version contains supplementary material available at 10.1186/s12954-025-01212-0.

## Background

Drug overdose caused more than 100,000 deaths annually in 2021–2023 [1]. Multiple factors have contributed to the growth in fatal and nonfatal overdose rates, including increased availability of highly potent illicitly manufactured fentanyl [2], polysubstance use [3], stigma [4], inadequate access and uptake of substance use disorder treatment [5], unemployment and other economic hardships [6], and mental illness and psychological distress that intersects with substance use disorders [7, 8]. Effective responses to overdose therefore must be multipronged and sensitive to the complex and diverse needs of people who use drugs (PWUD).

Comprehensive, community-based syringe services programs (SSPs) exemplify the type of flexible, person-centered approach necessary to address the overdose crisis. Harm reduction services provide safer drug use equipment, naloxone, and testing for communicable diseases in “low-threshold” settings, which have minimal requirements for service eligibility, and maintain client autonomy [9]. SSP utilization has been associated with a myriad of health benefits. Utilization of SSPs is associated with reduced human immunodeficiency virus (HIV) and hepatitis C virus (HCV) incidence, reductions in soft tissue infections and endocarditis, and increased linkage to substance use treatment and other health services such as HIV and HCV testing and treatment [10–13]. These benefits are in part because SSP access and utilization are associated with adoption of safer drug use practices, such as avoiding borrowing and reusing syringes and carrying naloxone [14–16]. Importantly, SSPs and other harm reduction venues, such as overdose prevention centers, are associated with decreased community overdose rates [17, 18]. Despite the success of SSPs, gaps in access to harm reduction resources such as naloxone and sterile syringes are widespread [19–21]. Many PWUD have inadequate access to comprehensive SSPs, particularly in rural and other settings where harm reduction is stigmatized or criminalized [20, 22]. Lack of access to SSPs leaves PWUD vulnerable to negative health outcomes, including infections and drug overdose [22].

Recently, phone-, internet-, mail-, and vending machine-based harm reduction services have become available and may have further reach for PWUD who are unable to access SSPs in-person [23–30]. Studies suggest these services can effectively deliver sterile syringes and overdose prevention and response education and are acceptable to PWUD [28, 31–33]. However, the psychosocial benefits derived from engaging with non-stigmatizing, in-person services may be difficult to replicate using virtual formats. For example, linkage to locally available services for food, housing, and other needed resources are often facilitated by in-person SSP services, but these supports may be less feasible when SSP services are delivered virtually or asynchronously.

This paper describes a new prospective cohort study that aims to characterize the influence of longitudinal engagement in harm reduction services on overdose risk behaviors among people who use illicit opioids and/or stimulants, the Exploration of Novel Harm Reduction Approaches to Increase Client Engagement (ENHANCE) Project. Specifically, we describe the community-engaged approach to developing the ENHANCE Project, the study protocol, and the planned analytic approach. ENHANCE data will be used to test the hypothesis that more frequent engagement with harm reduction services is associated with reductions in overdose risk behaviors. Secondarily, we hypothesize an explanatory mechanism for this relationship: harm reduction services help clients satisfy essential needs (e.g., food and healthcare access), which are associated with behavioral changes that reduce overdose risk. The results of the ENHANCE Project will therefore identify aspects of existing SSP services that are most critical to addressing the US overdose crisis.

## Methods

The ENHANCE Project was funded in September 2022 as part of the National Institutes of Health (NIH) Helping End Addiction Long-Term® (NIH HEAL Initiative®) Harm Reduction Research Network (HRRN). The research is conducted by a multi-site team at the University of Wisconsin-Madison, Tulane University, and Vivent Health, a community-based HIV service organization that provides syringe services through its LifePoint SSPs located in 10 cities in Wisconsin (Supplementary Fig. 1).

### Community leadership teams: community-engaged approach to developing the ENHANCE project

The community-engaged approach to developing the ENHANCE Project was created using several foundational principles from the National Harm Reduction Coalition [34]. These principles recognize the historical, social, and structural determinants of vulnerability to the negative impacts of drug use and the essential value of non-judgmental and non-coercive provision of services in SSPs [34]. By embodying these principles, SSPs empower their clients to support the safety and well-being of peers, which extends the reach of programming and health information beyond the SSP through trusted messengers [35]. A key principle guiding this study was the importance of creating programs and policies that reflect community and individual needs and that are shaped by PWUD.

To align with these foundational principles, the research team proposed and implemented a Community Leadership Team (CLT), composed of 16 people who use drugs, recruited from four Vivent Health LifePoint SSPs operated in Madison, Milwaukee, La Crosse, and Green Bay, Wisconsin. The CLT provided the opportunity to design and implement research that is reflective of community need and, “ensures that people who use drugs and those with a history of drug use routinely have a real voice in the creation of programs and policies designed to serve them” [34]. The creation of the CLT was led by prevention team members from Vivent Health LifePoint SSPs to reduce anticipated power imbalances between people who use drugs and academic researchers, while also empowering CLT members to make study design decisions [36, 37]. SSP staff at each project site invited 3–4 clients using opioids and/or stimulants that they perceived to be community leaders with a potential interest in overdose prevention based on their interactions with these clients during their in prevention services. SSP staff invited clients to participate in the CLT during face-to-face interactions at regular service appointments, and continued to recruit until they had secured commitment from four participants. The 16 CLT members are primarily white (94%), non-hispanic (88%), and identify as women (56%). Most members report using stimulants (88%) and heroin and/or fentanyl (63%).

**Fig. 1:**
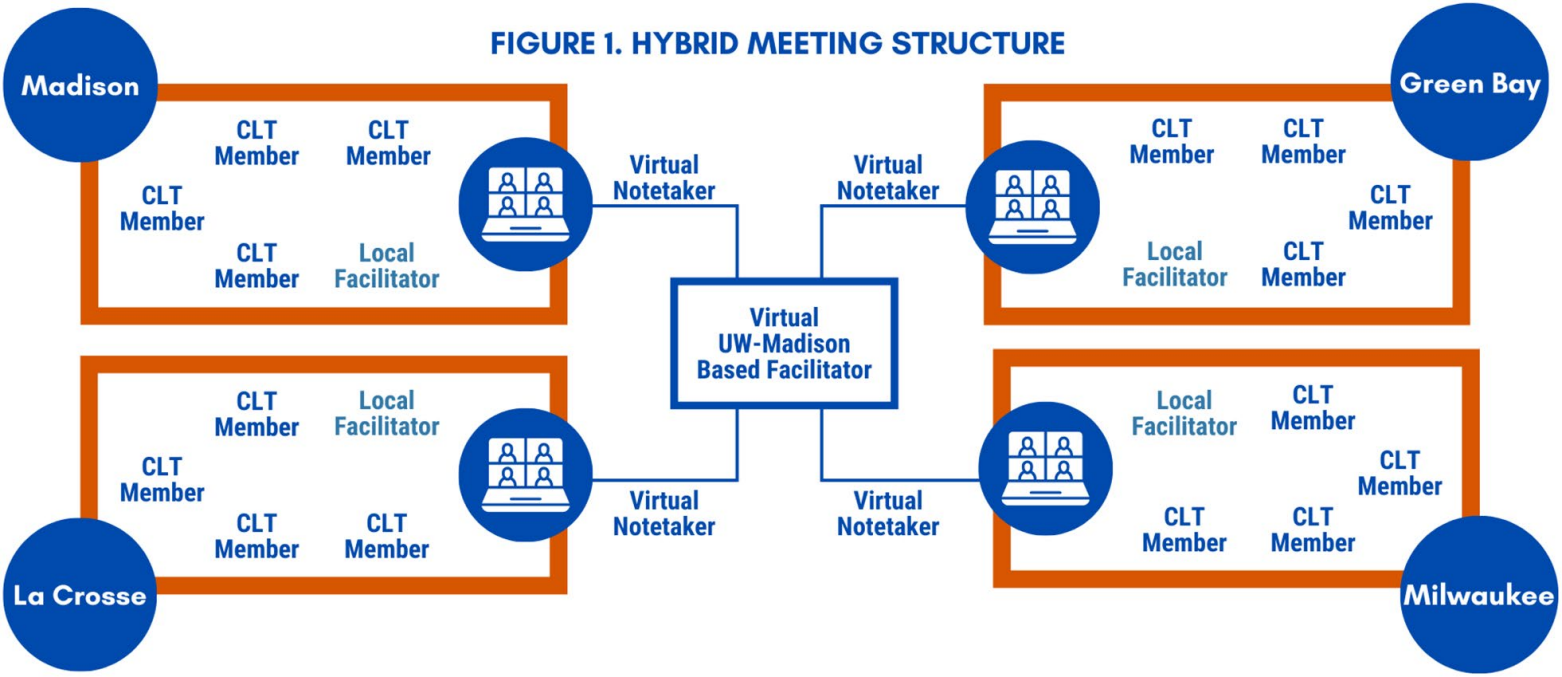
Community leadership team structure in 4 SSPs serving as study sites

CLT members participated in five meetings between June 2023 and January 2024 to inform the study protocol, data collection priorities, study advertisements, and other aspects of the study implementation plan. They received $50 remuneration for participating in each meeting. Because the CLT membership was spread across four offices in different cities, a hybrid meeting structure was adopted to facilitate local discussions while also collaborating as a statewide cohort (Figure 1). Members of the CLT gathered in their respective SSP office to share a meal. Each local group’s facilitator joined a multi-site virtual call, in which study staff introduced the focal topics of each meeting in a shared environment. Each site subsequently had a local discussion using a common facilitation guide prepared by the research team. A virtual notetaker captured themes of the conversation and reported key points at the close of each meeting when the four sites reconvened on the virtual call to learn about what the other sites had discussed. After each summary was provided, CLT members were asked to add anything that was missed or misrepresented by the notetaker.

### ENHANCE project recruitment, enrollment, and follow-up

ENHANCE Project enrollment opened in January 2024. CLT members were invited to enroll first and provide feedback on the enrollment process. They were also invited to recruit up to six additional members of their social networks to the study and received $10 remuneration for each enrolled person.

In February 2024, the ENHANCE Project began advertising the opportunity to enroll using flyers posted in SSP sites and mobile units, a study website, https://enhanceproject.wisc.edu/, and via word-of-mouth invitations from staff for clients receiving office-based or mobile van services at four Vivent Health SSPs serving as study sites (Supplementary Fig. 1). Enrolled clients can also invite up to three peers who use opioids and/or stimulants to complete the eligibility screener via referral coupons and are remunerated an additional $10 for each individual that they refer who enrolls in the study.

Interested clients complete a brief screening survey administered via Qualtrics to determine study eligibility. Inclusion criteria include (1) being 18 years or older and (2) self-reported use of either (a) illicit opioids and/or stimulants in the past 30 days by any mode, or (b) use of prescription opioid medications via smoking, snorting, or injecting in the past 30 days. Interested and eligible clients are invited to review the study procedures with SSP staff and watch videos that explain the study survey schedule, topics, and informed consent process. Interested clients then enroll by providing informed consent. From our four sites, we will recruit 125 clients in La Crosse and Milwaukee and 75 clients in Madison and Green Bay for a total sample size of *N* = 400. Target sample sizes by site were determined by SSP encounter volume.

**Fig. 2:**
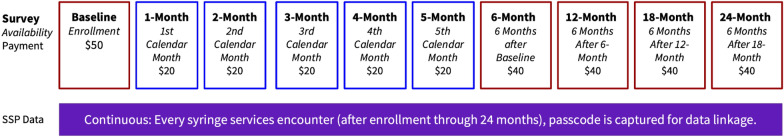
ENHANCE project participation timeline, surveys, and remuneration schedule

Enrolled clients complete an approximately 45-min baseline survey using Qualtrics and are remunerated $50 (Fig. 2). Clients are subsequently invited to complete 30-min monthly surveys using Qualtrics during months 1–5 and are remunerated $20 for each survey completed. Thereafter, clients are invited to complete 40-min surveys semi-annually during months 6, 12, 18, and 24 using Qualtrics and are remunerated $40 for each survey completed. Clients can select to be remunerated in two ways: (1) cash payments distributed by SSP staff from four Vivent Health offices serving as study sites, or (2) electronic text or email payments sent via an online renumeration platform, Tremendous, which allows payments to be redeemed as an online gift card or direct cash transfer. Multiple methods are used to invite and remind clients to complete surveys, including text messages, emails, and private messages on social media accounts. Qualtrics allows clients to access the surveys anywhere at any time, via their personal phones. Clients may also visit a SSP office or mobile van to use available study iPads to complete surveys.

Clients are able to check whether surveys are available using the study website. To do so, clients enter the component fields of a unique self-generated identification code created for the study (described further below) via a link on the study website. This unique code is verified against a Qualtrics directory, which is populated with each participant’s code during enrollment. An authentication workflow detects whether the unique code entered matches a listing in the directory, and if so, whether or not the client has already completed a survey in each of the first five months after enrollment. If they have not, they are redirected to the survey; if they have, they are reminded they can try again on the first of the next month. A similar process is used to route clients to open semi-annual surveys, which are available 6-months after enrollment, and subsequently, every 6-months after completion of the prior semi-annual survey. Study surveys include common data elements required for all HRRN sites as well as study-specific measures (described further below).

### Primary and secondary outcome measures: overdose risk behaviors and experiences

The primary outcome measures are self-reported frequency of several behaviors known to impact nonfatal and fatal overdose risk. Measures were conceptualized based on the Overdose Risk Behavior Scale (ORBS), which assesses past 30-day frequency of overdose risk behaviors related to opioid use. ORBS was developed in a population of veterans primarily using opioid analgesic medications, and subsequently modified and validated in a larger sample of people using illicit opioids [38, 39]. The primary outcomes include questions from several ORBS subscales, including the Solitary Opioid Use subscale (Cronbach’s alpha from validation study: 0.68), three polysubstance use subscales (i.e., Opioid/Alcohol [alpha: 0.83], Opioid/Benzodiazepine [0.64], Other Polysubstance Combinations [alpha: 0.84]), and the Injection Drug Use and Speedballing subscale (alpha: 0.56). As the US stimulant supply has become contaminated with illicitly manufactured fentanyl since the conceptualization and validation of ORBS [40–43], we additionally developed several parallel survey questions about stimulant-related risks from ORBS where applicable (e.g., solitary use of stimulant drugs). We additionally made slight modifications to the original ORBS where required HRRN common data elements represented only small variations in wording but overlapping intent with ORBS questions.

All primary outcome measure questions are assessed on the baseline and monthly surveys. Clients are asked to report on the number of days, in the past 30 days, that they used opioids or stimulants while alone, injected an opioid or stimulant, concomitantly used substances with known drug–drug interactions (i.e., opioids with stimulants, benzodiazepines, or alcohol), and used substances with known drug-drug interactions within a 6-hour period (i.e., opioids with stimulants, benzodiazepines, or alcohol). All measures will undergo psychometric analysis (i.e., calculation of Cronbach’s alpha and corrected item-total correlation) after baseline data are collected to inform analytic decisions about amending the originally conceptualized subscales from ORBS.

The secondary outcome is the number of nonfatal overdoses in the past 6 months, assessed on the baseline and semi-annual surveys. This is defined using HRRN common data elements for opioid overdose (survey question: “During the past 6 months, how many times did you overdose on drugs involving heroin, fentanyl or other opioids? Overdose means that you took enough of the drug that it caused a life-threatening reaction.”) and stimulant overamping (survey question: “In the past 6 months, how many times did you experience extreme mental or physical effects from using cocaine, methamphetamine, or other stimulant drugs that made you feel like you needed help [even if you didn’t seek care]? Symptoms may include chest pain, racing heart, nausea or vomiting, extreme sweating or high temperature, convulsions, seizures, cardiac arrest, or stroke. Mental health effects may include extreme anxiety, paranoia, or fear; hallucinations; and feeling stuck or frozen.”). We additionally ask clients to report on these events in the past 30 days on monthly surveys.

### Primary exposure: SSP utilization

Harm reduction services received at every client encounter at all 10 SSPs and their associated mobile vans within the Wisconsin Vivent Health network are collected anonymously in a cross-site database called Provide Enterprise. In collaboration with Vivent Health, we will capture confidential, but non-anonymous client administrative data for those who have provided informed consent for the ENHANCE Project using self-generated identification codes, an approach which has been successfully implemented in SSPs, including Vivent Health, and other contexts [44]. Self-generated identification codes are created at study enrollment by answering five questions, from which the following identification code is generated by Qualtrics: the first two letters of the client’s zodiac sign, the first three letters of the client’s eye color, the first two letters of the name of the city where the client was born, the first two letters of the client’s oldest parent’s first name, and the first two letters of the last school attended. Vivent Health staff at all 10 Wisconsin-based Vivent Health SSPs ask all SSP clients if they are participating in the study and, where affirmed, document the self-generated identification code in Provide Enterprise. The self-generated identification code is also used to access all study surveys via Qualtrics and will be used to link Provide Enterprise data on SSP encounters with survey data.

We will use linked SSP encounter data to create the primary independent variable for the ENHANCE Project primary analysis, which is the number of SSP encounters. Additionally, Vivent Health collects standardized data about each client encounter that we will use to describe services received. These data elements include the number of several types of supplies distributed (i.e., naloxone, syringes, smoking kits, fentanyl test strips, and xylazine test strips), whether the client was trained to respond to an opioid overdose, and the number of referrals for several off-site services provided, including medications for opioid use disorder, other substance use services (e.g., behavioral therapy), health insurance enrollment, housing and food assistance, mental health services, HCV or HIV treatment, and primary care.

### Mediators: satisfaction of essential needs and stigma

We will examine several potential mediators of the relationship between SSP engagement and overdose risk, including satisfaction of basic needs, satisfaction of psychological needs, and stigma. We hypothesize that engaging with SSPs may facilitate acquisition of essential needs and reduce overdose risk. We will assess satisfaction of basic needs using an HRRN common data element that assesses whether clients have been unable to access housing, food, medical care, transportation, or other needs. We will assess satisfaction of psychological needs using 12-items on psychological need satisfaction from the Basic Psychological Need Satisfaction and Frustration Scale [45]. We will also examine stigma as a mediator given that SSPs provide non-judgmental, person-centered services in a supportive environment, which may be critical to overdose risk reduction. We will measure enacted and internalized stigma using the Substance Use Stigma Mechanism Scale (Cronbach’s alpha for enacted stigma sub-scale: 0.90, Cronbach’s alpha for internalized stigma sub-scale 0.93) [46].

### Analytic approach

After the completion of data collection, we will test the association between SSP utilization frequency with overdose risk behavior frequency and occurrence of nonfatal overdose and overamping events. We propose to model associations of SSP utilization and study outcomes using zero-inflated Poisson or negative binomial marginal structural models. Models examining the outcome of overdose risk behavior frequency will use data from the baseline and monthly surveys to examine how SSP utilization in the month between surveys is associated with overdose risk behavior frequency reported in the subsequent month, incorporating a one-month lag period. We will use data collected over the entire 24 months to examine the association of SSP utilization over each 6-month period with nonfatal overdose occurrence or overamping events in the subsequent 6 months, again using a lagged approach.

To test the hypothesis that the mechanism by which SSP engagement reduces overdose risk behaviors is via helping clients meet basic and psychological needs and reducing stigma, we will use a causal mediation analysis approach that builds on our marginal structural models described above. We will again examine short-term (from monthly data) and longer-term (from semi-annual data) associations with lagged data.

All analyses will adjust for confounding using variables measured in all surveys. Potential confounders include sociodemographic characteristics, drug use history and behaviors (i.e., frequency, mode of use, substance use disorder severity, withdrawal, treatment), non-SSP sources for supplies (i.e., pharmacies, secondary exchange), safety strategies used (e.g., fentanyl or xylazine test strips), social support, mental health (e.g., depression), and criminal legal system involvement.

## Results

### Study design input from the CLT

The CLT influenced the study purpose, recruitment and retention plan, and other operational aspects of the ENHANCE Project (Table 1). CLT members suggested placing a strong emphasis on the purpose of the study—to save lives and prevent overdose—when recruiting potential clients, to encourage consistent study participation over the two-year follow-up period. CLT feedback on survey remuneration amounts and payment modalities resulted in significant increases to planned remuneration prior to beginning the study. The CLT also shared their priorities for survey topics, which included overdose, access to equipment, sex work, and requests for injection assistance. They also counseled the research team about how to approach sensitive topics in ways that minimize client discomfort or harm. Additionally, the CLT emphasized the importance of protecting the confidentiality and anonymity of people involved in the project.

**Table 1:** Discussion topics and key findings from each community leadership team meeting

Meeting number	Topics discussed	Findings, recommendations, and outcomes
1	● Project overview ● Marketing ● Recruitment sites and strategies (e.g., using mobile unit) ● Client engagement & retention	● Outreach in SSP is effective recruitment strategy ● Word of mouth is best way to reach people who do not visit SSP ● Interest in contributing to “greater good” and preventing overdose are motivators for study participation long-term ● Importance of privacy and confidentiality ● Brainstormed imagery for project logo and marketing materials
2	● Project branding (name, imagery, logo) ● Survey topics, questions, and importance ● Discussion of best way to handle sensitive topics on surveys	● Be straightforward in marketing materials about why the study is important (Help Prevent Overdose) ● Use “Project” instead of “Study”; Use “Client” instead of “Participant”—fosters a sense of belonging instead of being examined, while avoiding stigmatizing language ● Overdose, access to equipment, sex work, and requests for injection assistance are important survey topics ● Make sensitive questions optional, incorporate a content warning ahead of sensitive questions ● Importance of privacy given sensitive topics
3	● Review changes to project protocol and survey as result of CLT feedback ● Flyer & logo design ● Client flow through project activities ● Strategies for staying in touch with clients ● Remuneration	● Increase remuneration ● Payment by text/email link and option to transfer online payment to cash transfer apps or personal bank accounts is important (gift cards not desirable) ● Give clients a choice about payment method so they can select what works best for them ● Ability to do surveys anywhere will be helpful ● Provided feedback on project logo draft created by a CLT member
4	● Discussed revisions to project flow, payment amounts, and marketing materials from previous feedback	● Approved increased compensation proposal ● Confidence in self-generated identification codes as a method for maintaining privacy ● Flexible survey completion windows and alignment with calendar months will be helpful
5	● Revisited project purpose ● Gathered feedback on enrollment experience, including: ○ Scheduling ○ Collecting contact information ○ Consent ○ Self-generated identification code creation & use in SSP ○ Baseline survey ○ Payment ○ Referral coupons	● Consistency in enrollment day(s) important (i.e., one site always enrolls on Wednesdays) ● Consent and Project Overview videos were clear and easy to understand ● Suggestion to add more context around collecting contact information from clients ● Baseline survey was very long, hard to keep track of back-to-back questions asking about modes of use, suggestion to bold timeframe and mode of use ● Importance of field staff letting clients know that surveys after baseline are much shorter ● Questions about Xylazine were difficult to answer because members are unsure whether or not it is in their supply, add clarifying language to survey question, “Xylazine (that you knew was xylazine at the time of use)”

**Fig. 3:**
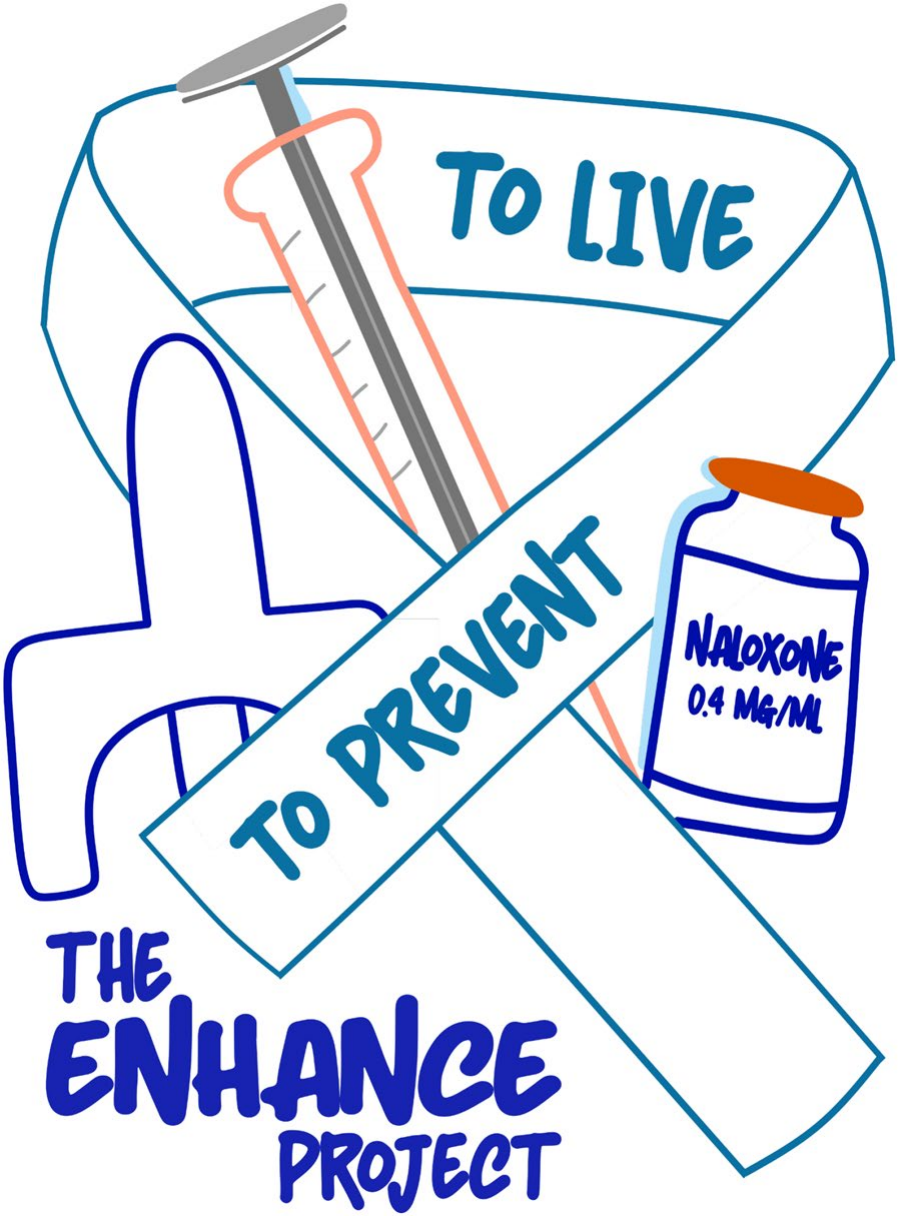
ENHANCE project study logo designed by a member of the community leadership team

Discussion about the stigma associated with traditional research language led the study team to rename the ENHANCE “study” the ENHANCE “project” and refer to “participants” as “clients.” CLT members expressed negative associations with the words “study” and “participant,” sharing that they associated these terms with thoughts that they were a “rat in a cage” or “under a microscope,” instead of the intended goal of the community working together with researchers to prevent overdose. Members of the CLT brainstormed imagery for a project logo, and one CLT member designed the study logo (Fig. 3). The CLT continues to meet regularly with the study team to review preliminary findings from the prospective cohort study and to conceptualize and design a pilot intervention focused on reducing overdose risk for those who are disengaged from or not able to access in-person harm reduction services, which is part of a subsequent aim of our study.

### Preliminary results from the first 125 ENHANCE project participants

Enrollment for the ENHANCE Project opened in January 2024 and will continue through March 2025. Sociodemographic characteristics from the first 125 clients enrolled from January–May 2024 suggest that clients predominantly identify as white race (66%) and male gender (63%) and report a median age of 40.5 years (Table 2). At enrollment, a majority (93%) had used a stimulant (methamphetamine, crystal meth, crack, or cocaine) in the past 30 days and 70% used heroin or fentanyl in the past 30 days. Approximately 22% reported personally experiencing an opioid overdose in the 6 months before enrollment and 38% experienced adverse effects after using stimulants, known as overamping. Overdose risk behaviors were commonly endorsed; participants reported that in the past 30 days, they used opioids while alone on an average of 9.9 days (standard deviation [SD]: 10.7), injected heroin on an average of 9.3 days (SD: 12.8), used cocaine and heroin at the same time on an average of 4.1 days (SD: 9.3), and used opioids and benzodiazepines within a 6-h period on an average of 4.8 days (SD: 9.4).

**Table 2:** Socio-demographic characteristics and primary outcomes for the first *N* = 125 ENHANCE project clients at enrollment

Characteristic	N (%)
Total	125
Age, Median (Range)	40.5 (23.0, 65.0)
*Race*	
American Indian or Alaska Native	9 (7.2)
Black	30 (24.0)
White	82 (65.6)
Prefer not to answer	4 (3.2)
Hispanic Ethnicity	13 (10.4)
*Gender*	
Male	79 (63.2)
Female	43 (34.4)
Transgender, gender-queer, nonbinary, questioning gender identity, or something else	3 (2.4)
High school/GED or higher education	106 (84.8)
Used heroin or fentanyl in the past 30 days	88 (70.4)
Used methamphetamine, crystal meth, crack, or cocaine in the past 30 days	116 (92.8)
Used prescription opiate pain relievers not as prescribed by smoking/vaping, snorting, or injecting in the past 30 days	36 (28.8)
Experienced an opioid overdose in the past 6 months	28 (22.4)
Overamped (adverse effect from taking stimulants) in the past 6 months	48 (38.4)
*Overdose risk behaviors in the past 30 days*	*Mean (SD) days*
Injected fentanyl	7.8 (12.0)
Injected heroin	9.3 (12.8)
Injected methamphetamine	5.4 (8.9)
Injected cocaine	3.6 (8.2)
Used methamphetamine and fentanyl concurrently	3.5 (8.3)
Used methamphetamine and heroin concurrently	2.0 (6.3)
Used cocaine and fentanyl concurrently	3.4 (9.0)
Used cocaine and heroin concurrently	4.1 (9.3)
Used fentanyl and xylazine concurrently	1.7 (6.0)
Used opioids and alcohol within a 6 h time period	4.7 (8.9)
Used opioids and benzodiazepines within a 6 h time period	4.8 (9.4)
Used opioids and any stimulant within a 6 h time period	11.2 (11.5)
Solitary opioid use	9.9 (10.7)
Solitary stimulant use	11.0 (11.7)

## Discussion

The ENHANCE Project, an ongoing, prospective cohort study, will provide critical insights about the benefits of harm reduction services, beyond the provision of supplies, for overdose risk reduction and prevention. In a subsequent phase of our grant, we will use the results from the cohort study and work with our CLT to design and pilot test an intervention for reducing overdose risk for people disengaged from or unable to reach brick-and-mortar or mobile unit harm reduction services. Leveraging the results of the cohort study will help us ensure that the most critical aspects of harm reduction services are adapted when applying new interventions to extend the reach of harm reduction services to under-served clients.

The ENHANCE Project will additionally provide longitudinal survey and SSP utilization data that is positioned to answer both our primary research questions about overdose risk as well as many secondary research questions. Regarding our primary analyses, our study will be one of the first to examine frequency of overdose risk behaviors longitudinally using several subscales of the ORBS, which will provide critical insights about why participants may have experienced or not experienced nonfatal overdose events they are also asked to self-report on all surveys. Relative to another recent study using the ORBS in an urban US sample [47], our sample appears to report solitary opioid use more frequently and concomitant use of opioids with other drugs or alcohol less frequently at baseline, which could have implications for risk of nonfatal and fatal overdose. Regarding secondary analyses, our team is collecting data on utilization and preferences for other harm reduction services outside of SSPs, such as virtual overdose monitoring services, naloxone vending machines, and mail-order services. Corresponding with areas highlighted as important by the CLT, we are capturing detailed information on issues such as solitary drug use and requests for assistance with injecting drugs using questions from a multi-site cohort consortium [48]. Moreover, we are collecting longitudinal data on drugs used, mode of use, substance use disorder treatment, criminal legal system involvement, and many other measures that are part of the common data elements for the HRRN, many of which were also priorities for the CLT.

### Limitations

We anticipate several potential challenges with the study implementation and analysis. First, loss to follow-up in prospective cohort studies involving clients actively using drugs can be substantial and result in missing survey data. We anticipated up to 30% attrition in sample size calculations based on prior studies our team has conducted with Vivent Health. To minimize loss to follow-up, we frequently survey clients in the first 6 months (i.e., monthly) and provide flexibility regarding the time intervals between surveys (i.e., monthly surveys are available anytime during the month, semi-annual surveys remain open 6-months after the last survey). We also provide multiple modalities for data collection and remuneration, with a mixture of in-office and remote access options to promote a client-centered experience. This may be particularly important for retaining clients who have stopped using drugs and wish to avoid the SSP. Another source of missing data may be from clients who unfortunately pass away from overdose or other causes during the study. At the conclusion of follow-up, we will explore identifying participants who have died and ascertaining causes of death through available national registries such as the National Death Index. Though it is unlikely we will be powered to examine fatal overdose as an outcome measure, these data may be helpful in interpreting our analyses of overdose risk behaviors and nonfatal overdose. Regarding measures, the ORBS scales used to conceptualize the primary outcomes had to be modified to incorporate risks related to overdose from stimulants contaminated with illicitly manufactured fentanyl and new substances, such as xylazine. Thus, we will assess psychometric properties and adjust sub-scales where appropriate in initial data cleaning and analyses. Finally, we anticipate that some CLT members may disengage from the project, at which time other interested clients who SSP staff believe to be a good fit will be approached to maintain representation of three to four clients per site.

## Conclusions

The ENHANCE Project will provide new insights about why and how client-centered, non-judgmental harm reduction services benefit PWUD and address the overdose crisis. The insights gleaned from this cohort study, which was designed in close collaboration with PWUD in Wisconsin and Wisconsin’s largest network of syringe services programs, will help to bolster existing SSP services as well as identify key components requiring adaptation as novel and flexible harm reduction tools, such as virtual overdose monitoring services, mail-based harm reduction, and others, are scaled and implemented.

## Supplementary Information

Supplementary material 1.

## Data Availability

The datasets generated and/or analyzed during the current study will become available at the conclusion of data collection and cleaning through the National Addiction & HIV Data Archive Program (NAHDAP, available at https://www.icpsr.umich.edu/web/pages/NAHDAP/index.html). Inquiries about access to data prior to depositing data on NAHDAP can be sent to the corresponding author for consideration.
