# Supplementary material for: Exploration of novel harm reduction approaches to increase client engagement (ENHANCE): protocol for a prospective cohort study
Source: Harm Reduct J. 2025 May 19;22(Suppl 1):81. doi: 10.1186/s12954-025-01212-0 (PMC12087053; doi:10.1186/s12954-025-01212-0)
Supplement: Supplementary file 1 — Supplementary material 1. [file 12954_2025_1212_MOESM1_ESM.docx]

**Supplementary Figure 1. Syringe Services Programs (SSPs) Serving as ENHANCE Project Study Sites and Additional SSPs Collecting SSP Utilization Data**

**
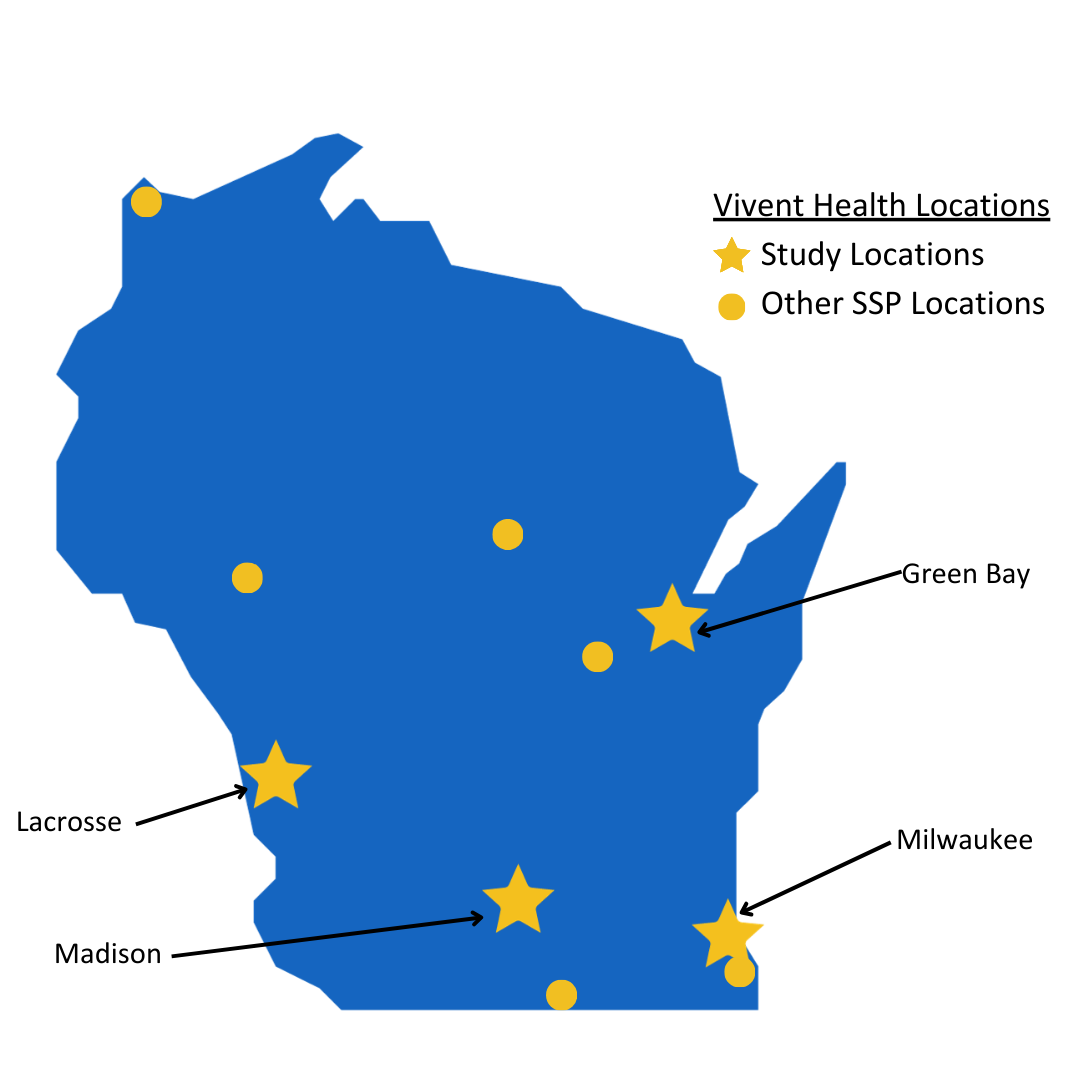
**
